# Residential proximity to major roadways and prevalent diabetes in older adults: a cross-sectional study based on CLHLS

**DOI:** 10.1186/s12877-026-08060-y

**Published:** 2026-07-28

**Authors:** Yong Li, Shu Feng, Huiying Liu, Xinzhu Shi, Nan Wang

**Affiliations:** 1 Central Hospital of Dalian University of Technology (Dalian Municipal Central Hospital), Dalian, China; 2 School of Public Health, Shenyang Medical College, Shenyang, China; 3 Department of Liaoning Province, Key Laboratory of Environment and Population Health of the Educational, Shenyang, China

**Keywords:** Residential proximity, Major roadways, Diabetes, CLHLS, Traffic-related environmental exposure, Older adults

## Abstract

**Background:**

Diabetes is a major global public health concern, and China bears a growing diabetes burden that calls for targeted prevention. High-income country studies link residential proximity to roadways with diabetes, yet evidence from developing countries is limited. This population-based analysis delivers robust evidence for environmental health interventions and age-friendly urban planning for Chinese older adults.

**Methods:**

We used nationally representative data from the Chinese Longitudinal Healthy Longevity Survey (CLHLS). After excluding those with missing values for key variables, 4,702 adults aged ≥ 65 years entered the complete-case primary analysis. Roadway proximity and diabetes status were self-reported. Logistic regression models calculated ORs and 95% CIs with sequential adjustment for demographic, lifestyle and health covariates. Stratified analyses and interaction tests assessed effect modification.

**Results:**

The sample included 4,702 participants (mean age 78.7 years; 55.1% men) with 13.9% diabetes prevalence. Fully adjusted ORs were 1.36 (95% CI:1.11–1.67) for residents < 100 m from major roadways and 1.31 (95% CI:1.03–1.66) for those 100–300 m away (reference group: > 300 m). A statistically significant overall trend test was obtained, yet the exposure–response relationship across distance strata was non-monotonic (*P* for trend < 0.05). A borderline interaction existed between roadway proximity and smoking (*P* for interaction = 0.044). Stratification showed a positive association among non-smokers (OR = 1.29, 95% CI:1.06–1.56) but no significant link in smokers (OR = 0.82, 95% CI:0.48–1.42). Given the marginal *P*-value and small smoking subgroup sample (wide CIs), this interaction needs cautious interpretation and verification in larger cohorts.

**Conclusion:**

This cross-sectional study found a significant association between proximity to major roadways and prevalent diabetes in Chinese older adults. Roadway proximity acts as a composite surrogate marker of traffic hazards, neighborhood socioeconomic deprivation and built environments, rather than merely traffic pollution or noise. These findings guide future environmental and health equity research on seniors. Longitudinal studies with objective exposure measurements are required to confirm our results and inform age-friendly urban design and geriatric health policies.

**Supplementary Information:**

The online version contains supplementary material available at https://doi.org/10.1186/s12877-026-08060-y.

## Background

Diabetes is a leading global cause of morbidity and mortality and has evolved into a severe public health crisis. According to data released by the International Diabetes Federation (IDF), the number of adults living with diabetes surpassed 500 million in 2024, with projections reaching nearly 900 million by 2050. The prevalence among adults aged 65 years and older stands at 23.72%, which is higher in middle-income countries compared with high-income and low-income nations [1]. As the world’s most populous nation, China bears a worsening diabetes epidemic: prevalence surged from below 1.0% in 1980 to 12.4% in 2018, creating substantial burdens for individuals, family caregivers, and the national healthcare system [2]. Data from China’s Seventh National Population Census indicated that the population aged 60 years and older hit 260.4 million (18.7% of the total population) in 2020, and roughly 30% of this elderly cohort suffered from diabetes at that time. Acute and chronic complications stemming from suboptimal glycemic control have emerged as key threats to the health and longevity of older adults [3].

Diabetes onset stems from the interplay of genetic susceptibility and modifiable lifestyle factors [4–6]. Chronic exposure to adverse residential environments, including traffic-derived pollutants, may raise diabetes risk by inducing sustained systemic inflammation and metabolic dysfunction [7–9]. Previous epidemiological studies have detected an association between residential proximity to major roadways and prevalent diabetes, yet robust evidence from Chinese populations remains limited.

Most published analyses exploring associations between residential proximity to major roadways and type 2 diabetes have been conducted in Western populations, with consistent evidence showing that proximity to high-traffic corridors significantly elevates the odds of type 2 diabetes [10, 11]. Rapid urbanization and rising motor vehicle volumes over recent decades have worsened traffic-related pollution across low- and middle-income countries, magnifying health risks for residents living close to roadways. However, few epidemiological investigations have quantified the association between roadway proximity and diabetes prevalence in such contexts. Against this backdrop, further evidence is warranted to clarify this relationship among Chinese older adults.

This cross-sectional analysis drew on data from the Chinese Longitudinal Healthy Longevity Survey (CLHLS), a large, nationally representative cohort that follows older adults nationwide in China. We combined information on residential proximity to major roadways, self-reported diabetes status, and an extensive panel of covariates. We adjusted for socioeconomic and lifestyle-related factors to evaluate associations between roadway proximity and prevalent diabetes among Chinese adults aged ≥ 65 years. The results yield important public health insights into environment–diabetes associations among older adults in developing countries.

## Methods

### Study participants

**Fig. 1:**
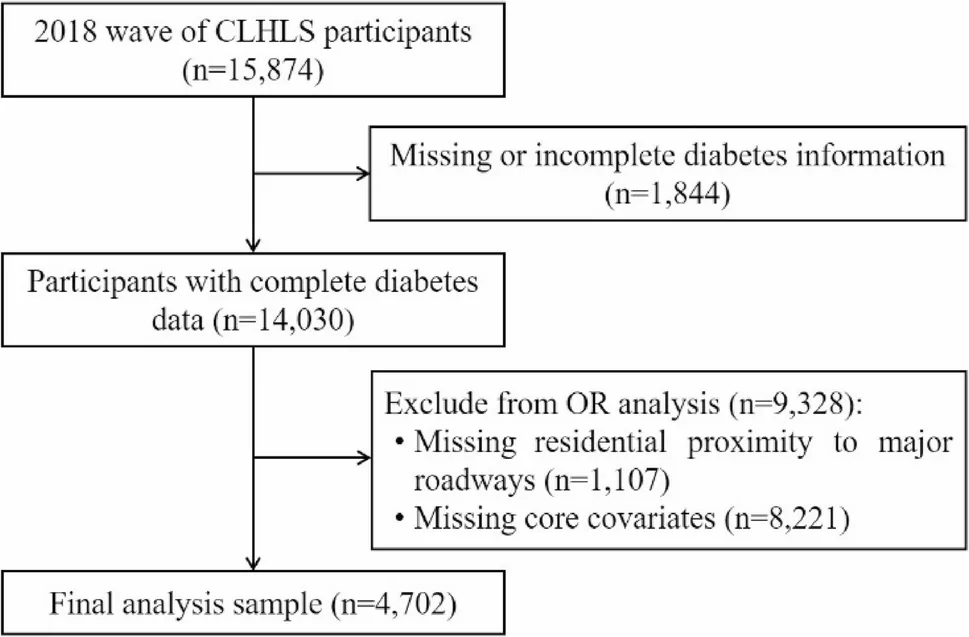
Flowchart illustrating participant selection for the study using data from the Chinese Longitudinal Healthy Longevity Survey (CLHLS). Figure legend: The full CLHLS cohort contained 15,874 participants. Participants with missing diabetes outcome data, missing roadway proximity exposure data, or incomplete core covariate records were sequentially excluded. The final complete-case analytical sample included 4,702 individuals

The Chinese Longitudinal Healthy Longevity Survey (CLHLS), launched in 1998, is a nationally representative longitudinal survey of Chinese older adults. It employs multi-stage cluster sampling across 23 regions, which together account for over 85% of China’s total population. A detailed description of the survey has been published elsewhere [12], and the CLHLS questionnaires used in this study are standardized and validated in prior publications. We adopted the 2018 CLHLS wave, which was the first round to include self-reported distance to major roadways. The initial sample comprised 15,874 participants aged ≥ 65 years. After excluding participants with missing diabetes status (*n* = 1,844), roadway proximity data (*n* = 1,107), or key covariates (*n* = 8,221), the final analytical sample comprised 4,702 adults aged ≥ 65 years (Fig. 1).

The CLHLS obtained ethical approval from the Research Ethics Committees of Peking University and Duke University (IRB No. 00001052–13074). All participants or their proxy respondents provided written informed consent prior to the interviews.

### Residential proximity to major roadways

Residential proximity to major roadways was self-reported by participants or proxy respondents. The CLHLS questionnaire lacks official standardized road classification criteria. Consistent with prior environmental analyses using CLHLS data [13], we defined major roadways operationally as dual four-lane carriageways. Nevertheless, participants’ self-reported distance assessments relied on their subjective judgment of major roads, which may not fully match this study’s operational definition.

Respondents estimated their distance to the nearest major roadway, which was grouped into five predefined intervals: < 50 m, 50–100 m, 101–200 m, 201–300 m, and > 300 m. For the primary logistic regression models, these five strata were collapsed into three exposure categories: < 100 m, 100–300 m, and > 300 m, with the > 300 m band designated as the reference group. No geocoding or on-site objective measurements were accessible to verify self-reported distance values, and proxy responses were allowed for participants with cognitive or physical disabilities.

### Assessment of diabetes

The primary outcome was prevalent type 2 diabetes, ascertained using self-reported physician-confirmed diagnoses from the CLHLS. Trained interviewers administered a standardized chronic disease module containing the question: “Has a doctor ever told you that you have diabetes?” Three response choices were provided: “yes”, “no”, and “do not know”. Respondents selecting “yes” were defined as diabetic, those answering “no” formed the non-diabetic reference group, and participants with missing or “do not know” responses were excluded to reduce misclassification bias. No on-site blood glucose or glycated hemoglobin (HbA1c) assays were performed during CLHLS fieldwork, so case identification for diabetes relied entirely on participants’ self-reported formal clinical diagnostic history.

### Covariates

A priori confounders were selected for regression models examining roadway proximity and prevalent diabetes, covering demographic, socioeconomic, lifestyle, and health factors: age (continuous); sex (male/female); educational level (no school vs. ≥ 1 year); residential location (city/town vs. rural); marital status (married vs. other categories, where others included widowed, separated, divorced or never-married participants); cohabitation status (yes/no); annual household income (≤ 10,000 yuan, 10,001–50,000 yuan, > 50,000 yuan); smoking status (yes/no); alcohol consumption (yes/no); regular physical activity (yes/no); body mass index (BMI, continuous); hypertension (yes/no); activity limitation (yes/no); self-reported quality of life (good/very good vs. average/poor/very poor); depressive symptoms (yes/no); anxiety symptoms (yes/no); cognitive impairment (yes/no).

Depressive symptoms were measured with the 10-item Center for Epidemiologic Studies Depression Scale (CES-D-10), using a cutoff score of ≥ 10 [14, 15]. Anxiety symptoms were screened via the 7-item Generalized Anxiety Disorder Scale (GAD-7), with a score of ≥ 5 set as the threshold for anxiety symptoms [16, 17]. Cognitive function was assessed using a 13-item CLHLS cognitive battery evaluating orientation, memory, attention, calculation, and language. Each item received a score of 0 (incorrect response) or 1 (correct response), generating a total score ranging from 0 to 30; higher total scores corresponded to better cognitive performance. Consistent with prior CLHLS research, cognitive impairment was defined by a total score < 24 [18, 19].

### Statistical analysis

#### Baseline characteristics

Baseline characteristics were summarized across three roadway proximity groups. Continuous variables were presented as mean ± standard deviation (SD), while categorical variables were reported as frequencies and percentages. Intergroup disparities were assessed via one-way ANOVA for continuous variables and χ² tests for categorical variables. Furthermore, among the 14,030 participants with complete diabetes status at baseline, we compared baseline features between individuals retained in the complete-case analysis and those excluded owing to missing covariates or roadway proximity data.

#### Primary multivariable logistic regression models

To assess the cross-sectional association between residential proximity to major roadways and prevalent diabetes, we built five logistic regression models with stepwise covariate adjustment. The crude model included no covariates. Model 1 was adjusted for age and sex only. Model 2 was additionally adjusted for residential location, educational level, marital status, smoking status, alcohol consumption, regular physical activity, and BMI. Model 3 further incorporated annual household income, cohabitation status, and self-reported quality of life. Model 4 (the primary fully adjusted model) was additionally adjusted for depressive symptoms, anxiety symptoms, cognitive impairment, activity limitation, and hypertension.

Supplementary Model 5 excluded five health-related indicators from Model 4: hypertension, depressive symptoms, anxiety symptoms, activity limitation, and self-reported quality of life. This model was designed to explore their potential role as mediating factors in the association between roadway proximity and diabetes, although this approach cannot substitute for formal mediation analysis. Although these indicators were predefined as confounders for the primary analysis, formal mediation analysis could not be conducted, as the cross-sectional design precludes verification of temporal ordering between roadway proximity, intermediate health conditions, and diabetes onset [20, 21].

#### Subgroup and interaction analyses

Roadway proximity was dichotomized (≤ 100 m vs. > 100 m) for stratified logistic regression across predefined effect modifiers: age (< 85 vs. ≥ 85), sex, residential location, educational level, annual household income (≤ 50,000 vs. > 50,000 yuan), BMI (< 24 vs. ≥ 24 kg/m²), smoking status, and alcohol consumption. Multiplicative interaction terms were constructed to test effect modification. All stratified regression models were adjusted for the full covariate set outlined in the Methods section, excluding the respective stratification variable from each model. The 85-year age cutoff and 24 kg/m² BMI threshold were selected in line with widely accepted Chinese standards for geriatric grouping and overweight classification [22, 23].

#### Sensitivity analyses

We performed seven pre-specified sensitivity analyses built on Model 4 to verify the robustness of the core association. First, we excluded participants with severe cognitive impairment (score < 18) or dementia to reduce recall bias [24]. Second, we omitted respondents with activities of daily living (ADL) limitations to mitigate potential reverse causality. Third, we removed participants diagnosed with malignant tumors to rule out confounding driven by severe chronic illness. Fourth, we fitted a logit-linked generalized linear model (GLM) to recalculate odds ratios (ORs) [25]. Fifth, we adopted two fully conditional specification multiple imputation (FCS-MI) strategies to address missing data in accordance with established methodological guidelines [26]. Model 6 (conventional FCS-MI) imputed all variables for the full baseline cohort (*n* = 15,874, the complete CLHLS sample prior to any exclusions), whereas Model 7 (outcome-fixed FCS-MI) only imputed exposure and covariate data among participants with confirmed complete diabetes status (*n* = 14,030). Full details of the imputation workflow, including model setup and convergence diagnostic metrics, are documented in Additional file 1 (Supplementary Methods). Sixth, we restricted the analysis to urban and township residents to evaluate confounding related to urban–rural disparities. Seventh, we re-fitted regression models using the original five fine-grained distance strata to explore granular exposure–response relationships.

All statistical tests were two-tailed, with *P* < 0.05 set as the threshold for statistical significance. All analyses were conducted using IBM SPSS Statistics 26.0.

## Results

### Baseline characteristics

Table 1 presents participants’ baseline characteristics. The full analytical sample had a mean age of 78.7 years (SD = 9.8); 55.1% were male, 61.1% lived in city or town areas, and the overall diabetes prevalence was 13.9%. Compared with those living closer to major roadways, participants living farther from roadways were more likely to dwell in rural settings, have lower educational levels and annual household income, and engage in smoking and alcohol consumption. This group also reported less regular physical activity and a lower prevalence of hypertension.

**Table 1:** Baseline characteristics of participants stratified by residential proximity to major roadways

Characteristics	Residential proximity to major roadways (m)				*P*-value
	< 100	100–300	> 300	Total	
*N*	1,739	975	1,988	4,702	
Age, years, mean (SD)	78.5 (9.7)	79.1 (10.0)	78.7 (9.8)	78.7 (9.8)	0.223
Sex, *n* (%)					0.107
Male	924 (53.1)	546 (56.0)	1,122 (56.4)	2,592 (55.1)	
Female	815 (46.9)	429 (44.0)	866 (43.6)	2,110 (44.9)	
Residential location, *n* (%)					< 0.001
City/Town	1,185 (68.1)	703 (72.1)	983 (49.4)	2,871 (61.1)	
Rural	554 (31.9)	272 (27.9)	1,005 (50.6)	1,831 (38.9)	
Educational level, *n* (%)					0.001
No school	424 (24.4)	201 (20.6)	533 (26.8)	1,158 (24.6)	
≥ 1 year	1,315 (75.6)	774 (79.4)	1,455 (73.2)	3,544 (75.4)	
Cohabitation status, *n* (%)					0.227
Yes	1,472 (84.6)	801 (82.2)	1,657 (83.4)	3,930 (83.6)	
No	267 (15.4)	174 (17.8)	331 (16.6)	772 (16.4)	
Self-reported quality of life, *n* (%)					0.032
Very good, good	1,254 (72.1)	734 (75.3)	1,505 (75.7)	3,493 (74.3)	
Average, bad, very bad	485 (27.9)	241 (24.7)	483 (24.3)	1,209 (25.7)	
Depressive symptoms, *n* (%)					0.891
Yes	590 (33.9)	338 (34.7)	672 (33.8)	1,600 (34.0)	
No	1,149 (66.1)	637 (65.3)	1,316 (66.2)	3,102 (66.0)	
Anxiety symptoms, *n* (%)					0.577
Yes	167 (9.6)	101 (10.4)	182 (9.2)	450 (9.6)	
No	1,572 (90.4)	874 (89.6)	1,806 (90.8)	4,252 (90.4)	
Cognitive impairment, *n* (%)					0.386
Yes	102 (5.9)	60 (6.2)	138 (6.9)	300 (6.4)	
No	1,637 (94.1)	915 (93.8)	1,850 (93.1)	4,402 (93.6)	
Smoking status, *n* (%)					0.004
Yes	330 (19.0)	154 (15.8)	416 (20.9)	900 (19.1)	
No	1,409 (81.0)	821 (84.2)	1,572 (79.1)	3,802 (80.9)	
Alcohol consumption, *n* (%)					0.045
Yes	306 (17.6)	176 (18.1)	410 (20.6)	892 (19.0)	
No	1,433 (82.4)	799 (81.9)	1,578 (79.4)	3,810 (81.0)	
Regular physical activity, *n* (%)					< 0.001
Yes	852 (49.0)	438 (44.9)	814 (40.9)	2,104 (44.7)	
No	887 (51.0)	537 (55.1)	1,174 (59.1)	2,598 (55.3)	
Activity limitation, *n* (%)					0.133
Yes	349 (20.1)	197 (20.2)	448 (22.5)	994 (21.1)	
No	1,390 (79.9)	778 (79.8)	1,540 (77.5)	3,708 (78.9)	
Annual household income (Yuan), *n* (%)					< 0.001
≤ 10,000	410 (23.6)	204 (20.9)	680 (34.2)	1,294 (27.5)	
10,001–50,000	548 (31.5)	276 (28.3)	695 (35.0)	1,519 (32.3)	
> 50,000	781 (44.9)	495 (50.8)	613 (30.8)	1,889 (40.2)	
Marital status, *n* (%)					0.596
Married	1,046 (60.1)	593 (60.8)	1,228 (61.8)	2,867 (61.0)	
Other categories	693 (39.9)	382 (39.2)	760 (38.2)	1,835 (39.0)	
BMI (kg/m^2^), mean (SD)	23.6 (5.9)	23.4 (3.9)	23.3 (8.1)	23.4 (6.6)	0.216
Hypertension, *n* (%)					0.001
Yes	831 (47.8)	511 (52.4)	896 (45.1)	2,238 (47.6)	
No	908 (52.2)	464 (47.6)	1,092 (54.9)	2,464 (52.4)	
Diabetes					< 0.001
Yes	279 (16.0)	165 (16.9)	209 (10.5)	653 (13.9)	
No	1,460 (84.0)	810 (83.1)	1,779 (89.5)	4,049 (86.1)	

^a^ Depressive symptoms were defined as a CES-D-10 total score of ≥ 10

^b^ Anxiety symptoms were defined as a GAD-7 total score of ≥ 5

^c^ Cognitive impairment was defined as a total cognitive function score of < 24

^d^ “Other categories” includes widowed, separated, divorced, and never-married participants

^e^ *BMI* Body mass index

### Association between residential proximity to major roadways and prevalent diabetes

Logistic regression was used to evaluate cross-sectional associations between residential proximity to major roadways and prevalent diabetes, with participants residing > 300 m from roadways serving as the reference group. In the unadjusted model, residents living < 100 m and those 100–300 m away exhibited elevated ORs of 1.63 (95% CI: 1.34–1.97) and 1.73 (95% CI: 1.39–2.16), respectively. Following full covariate adjustment (Model 4), effect estimates attenuated to 1.36 (95% CI: 1.11–1.67) and 1.31 (95% CI: 1.03–1.66) (Table 2). The overall trend test across ordered distance strata reached statistical significance (*P* < 0.05), yet the exposure–response pattern was non-monotonic: the association was slightly attenuated in the 100–300 m subgroup relative to participants living within 100 m, with no steady graded elevation as residential distance to roadways decreased.

**Table 2:** Associations between residential proximity to major roadways and prevalent diabetes

Model	Residential proximity to major roadways (m), OR (95% CI)			*P* for trend
	< 100	100–300	> 300	
Unadjusted crude model	1.63 (1.34, 1.97)	1.73 (1.39, 2.16)	Ref.	< 0.001
Model 1	1.61 (1.33, 1.95)	1.75 (1.40, 2.18)	Ref.	< 0.001
Model 2	1.37 (1.12, 1.67)	1.42 (1.13, 1.78)	Ref.	0.003
Model 3	1.31 (1.07, 1.60)	1.33 (1.06, 1.68)	Ref.	0.011
Model 4	1.36 (1.11, 1.67)	1.31 (1.03, 1.66)	Ref.	0.004

Crude model: no covariates adjusted

Model 1 was adjusted solely for age and sex

Model 2 additionally adjusted for residential location, educational level, marital status, smoking status, alcohol consumption, regular physical activity, and BMI

Model 3 further incorporated annual household income, cohabitation status, and self-reported quality of life

Model 4 (the primary fully adjusted model) additionally adjusted for depressive symptoms, anxiety symptoms, cognitive impairment, activity limitation, and hypertension

*Abbreviation*: *OR* Odds ratio, *CI* Confidence interval, *Ref*. Reference group

Additional file 2 (Supplementary Table S1) displays outputs from Model 5. This specification removed five health-related variables (hypertension, depressive symptoms, anxiety symptoms, activity limitation, and self-reported quality of life) from the fully adjusted primary model (Model 4) to preliminarily probe potential mediating pathways. The highly consistent ORs and 95% CIs generated by Model 4 and Model 5 suggested minimal mediating contributions from these five health factors. Importantly, this simplified model exclusion strategy is not a replacement for rigorous formal causal mediation analysis. Due to the cross-sectional study design, robust causal inferences about these mediating pathways cannot be established.

### Subgroup analyses and effect modification

**Fig. 2:**
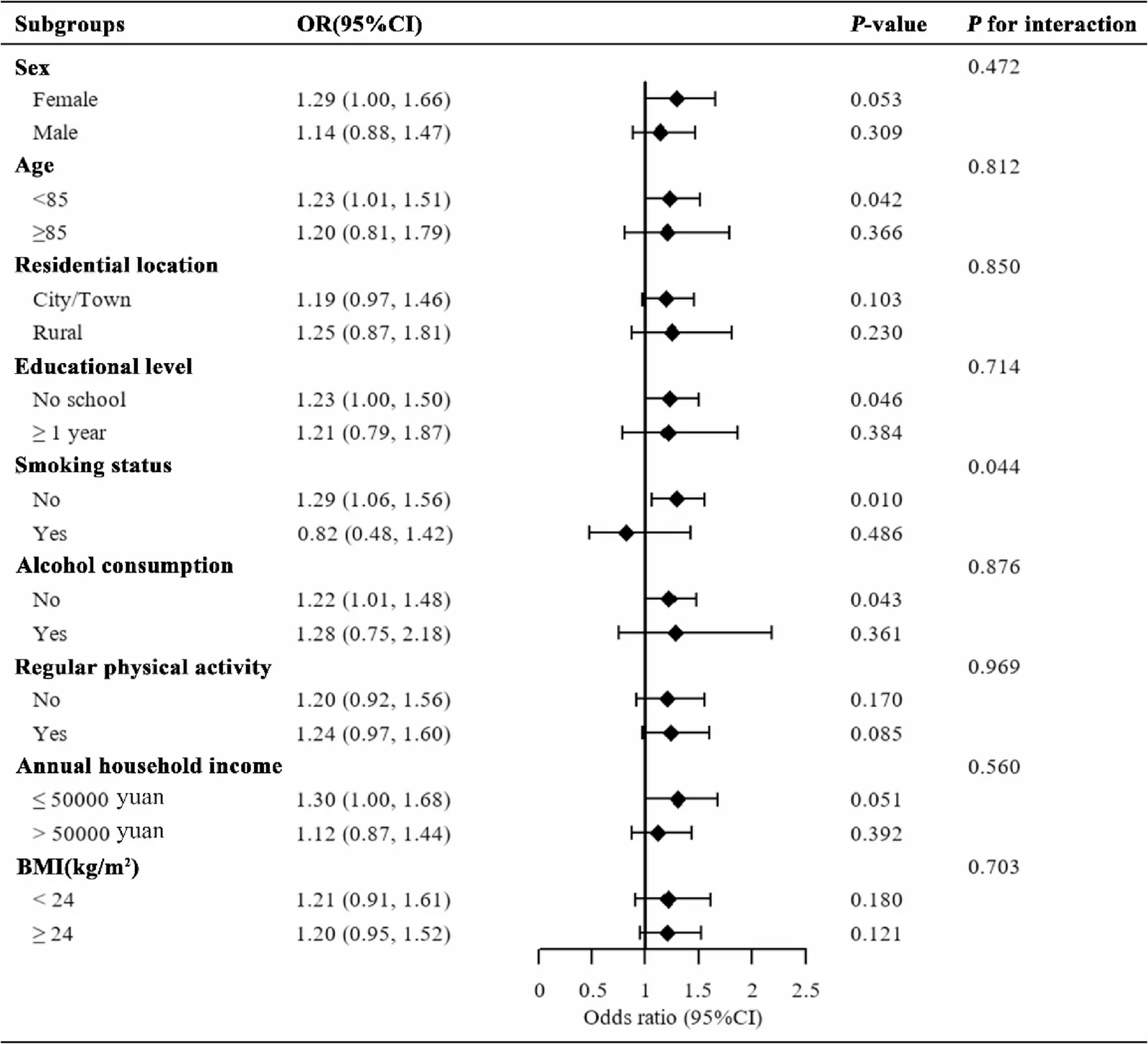
Forest plot illustrating associations between residential proximity to major roadways (< 100 m versus ≥ 100 m) and prevalent diabetes, stratified by core covariates. Figure legend: All stratified regression models were adjusted for all covariates listed in the Statistical Analysis subsection of Methods, excluding the respective stratification variable. The OR compares the odds of prevalent diabetes for participants living < 100 m from major roadways against those residing ≥ 100 m away. *P* for interaction values indicate the statistical significance of effect modification across subgroups. Abbreviations: OR, Odds ratio; CI, Confidence interval

Stratified subgroup analyses and multiplicative interaction tests were performed across predefined effect modifiers: age, sex, residential location, educational level, smoking status, alcohol consumption, regular physical activity, annual household income, and BMI (Fig. 2). The positive association between roadway proximity and prevalent diabetes showed comparable effect magnitudes across most subgroups.

A borderline multiplicative interaction between roadway proximity (< 100 m vs. ≥ 100 m) and smoking status was identified (*P* for interaction = 0.044). This marginal effect modification signal requires cautious interpretation, since interaction testing demands a larger sample to attain adequate statistical power. Stratified regression outputs revealed heterogeneous associations stratified by smoking status: non-smokers living within 100 m of major roadways had higher odds of diabetes (OR = 1.29, 95% CI: 1.06–1.56, *P* < 0.05), while no statistically significant association was seen among smokers (OR = 0.82, 95% CI: 0.48–1.42, *P* > 0.05). The broad confidence interval for the smoking subgroup indicates limited precision of effect estimation, stemming from the relatively small count of smokers in this elderly sample.

### Sensitivity analysis

We observed systematic disparities in baseline characteristics between participants retained in the primary complete-case analysis (*n* = 4,702) and individuals excluded owing to missing data (*n* = 9,328), with *P* < 0.001 across all intergroup comparisons (Additional file 3 [Supplementary Table S2]). This gap implies potential selection bias arising from substantial missing values. All seven pre-specified sensitivity analyses verified steady, statistically significant positive associations between roadway proximity and prevalent diabetes. The sensitivity tests covered sequential exclusion of participants with severe cognitive impairment, ADL limitations, or malignant tumors; logit-linked Poisson regression; two distinct FCS-MI strategies; restricted analysis to urban and township residents; and models built on the five fine-grained distance bands. All sensitivity tests generated consistent effect estimates (Table 3, Additional file 4 [Supplementary Table S3], Additional file 5 [Supplementary Table S4]). FMI values lower than 0.3 demonstrated that missing data produced minimal distortion to the core effect estimates.

**Table 3:** Sensitivity analyses of associations between residential proximity to major roadways and prevalent diabetes

Model	Residential proximity to major roadways (m), OR (95% CI)			*P* for trend
	< 100	100–300	> 300	
Model A	1.35 (1.10, 1.67)	1.29 (1.02, 1.64)	Ref.	0.005
Model B	1.43 (1.15, 1.78)	1.32 (1.02, 1.70)	Ref.	0.001
Model C	1.41 (1.15, 1.74)	1.32 (1.04, 1.69)	Ref.	0.001
Model D	1.35 (1.09, 1.66)	1.29 (1.01, 1.63)	Ref.	0.004

Model A was constructed to exclude participants with severe cognitive impairment and dementia

Model B was constructed to exclude participants unable to perform activities of daily living (ADL)

Model C was constructed to exclude participants diagnosed with cancer

Model D adopted a logit-linked generalized linear model (GLM) with binomial distribution to model binary prevalent diabetes outcome

All sensitivity models (A, B, C, D) were adjusted for the identical covariate set of the primary fully adjusted Model 4

*Abbreviation*: *OR* Odds ratio, *CI* Confidence interval, *Ref*. Reference group

## Discussion

After full covariate adjustment, this cross-sectional analysis of 4,702 Chinese older adults identified a stable positive association between residential proximity to major roadways and prevalent diabetes. A statistically significant overall trend test was detected across distance strata, yet the exposure–response curve followed a non-monotonic shape. A borderline interaction with smoking status was identified: a statistically significant association was only evident among non-smokers. This stratified result is exploratory, as the smoking stratum had a small sample size, which resulted in low estimation precision and broad confidence intervals, restricting statistical power to detect an association. All sensitivity analyses validated the robustness of these core associations.

The stable association, validated across multiple sensitivity tests, has implications for older populations. Older adults are more susceptible to environmental hazards owing to age-related declines in physiological reserve [27], higher comorbidity burden [28], and polypharmacy that disrupts metabolic homeostasis [29]. Meanwhile, older adults spend more time at home than younger groups, yielding greater cumulative residential environmental exposure over time. Against China’s dual backdrop of rapid urbanization and population aging, even modest roadway–diabetes associations may translate into a substantial population-level disease burden. These findings highlight the value of age-friendly urban planning and community diabetes surveillance in urbanizing developing regions. Clinically, geriatricians and community health practitioners may incorporate residential environmental context as an auxiliary contextual indicator when evaluating diabetes-related profiles among older adults, especially in rapidly urbanizing areas. Mitigating environmental health disparities in metabolic diseases requires cross-sector collaboration integrating urban planning, traffic management, and community geriatric health promotion.

We then explore plausible biological and contextual pathways that may explain the observed association. Our results partially align with prior Western cohort studies documenting positive roadway–diabetes associations [30, 31], while two earlier analyses reported null associations [32, 33]. Roadway proximity correlates with traffic-related air pollutants and chronic noise, both of which are linked to impaired glucose homeostasis [34–36]. Socioeconomic and built environment attributes may also contribute to this relationship: residents living close to major roadways typically have limited green space, low walkability, restricted access to medical services, and elevated neighborhood deprivation [37–40]. These contextual features correlate with diabetes status and could act as mediators or confounders in the observed relationship. Sensitivity analyses indicated the association was not attributable merely to urban–rural disparities or how exposure strata were defined. In the absence of objective on-site monitoring, we are unable to disentangle independent effects of air pollution and noise; roadway proximity serves as a composite residential indicator instead of a precise single traffic exposure measurement. The detected association likely arises from overlapping environmental, behavioral, and socioeconomic correlates, rather than an effect originating exclusively from traffic.

Comparisons of outputs from Model 4 and Model 5 offer further clues regarding plausible mediating pathways. The nearly identical effect estimates derived from the fully adjusted model and its reduced specification indicate that these five health-related factors exerted negligible mediating contributions within this cross-sectional sample. However, the cross-sectional study design precludes rigorous formal causal mediation modeling; thus, firm inferences on intermediate pathways cannot be established [20, 21].

The observed heterogeneity across smoking subgroups remains exploratory and merits cautious interpretation. This pattern may be partially attributable to the limited sample volume and broad confidence intervals among smokers, yielding low-precision effect estimates. An alternative explanation points to survivor bias, through which metabolically vulnerable smokers drop out of the elderly population over time [41]. This well-documented “healthy smoker effect” has been widely reported in older adult cohorts [42]. Moreover, biological interplay between tobacco consumption and traffic-derived pollutants is presumed intricate, and selective survival within cross-sectional elderly samples may introduce additional confounding [43]. In light of these methodological and biological complexities, larger-scale mechanistic and longitudinal investigations are required prior to drawing robust definitive inferences.

This study carries several key limitations. First, the cross-sectional study design prohibits confirmation of the temporal ordering between residential roadway exposure and diabetes onset, precluding robust causal inferences. Second, both exposure status and diabetes outcomes were ascertained solely via self-report, without verification from laboratory testing or official medical records. The CLHLS dataset does not contain standardized roadway classification metrics, and geocoding coordinates were unavailable to validate participants’ self-reported residential distance to major roads. Recall bias and subjective measurement error could yield exposure misclassification. Though proxy interviews boosted response rates among frail older adults, this approach may have added further measurement inaccuracies. Self-reported diabetes status tends to underestimate true population prevalence; this form of non-differential misclassification would shift effect estimates toward the null, indicating our observed associations are conservative [44]. Third, over half of the full baseline cohort was excluded owing to incomplete covariate data, which could introduce selection bias. Supplementary comparisons in Additional file 3 (Supplementary Table S2) reveal marked disparities in baseline characteristics between retained and excluded participants. Two FCS-MI multiple imputation specifications mitigated this concern and reproduced consistent association patterns, yet residual selection bias may still limit the external generalizability of our results. Fourth, unmeasured neighborhood deprivation, housing conditions, and healthcare access generate residual confounding that cannot be fully accounted for by individual socioeconomic indicators and urban–rural stratification. Roadway proximity encapsulates multiple correlated environmental inputs—including traffic-derived air pollutants, neighborhood socioeconomic disadvantage, and suboptimal built environment features—so we could not disentangle independent associations uniquely attributable to traffic-related exposures from these intertwined contextual factors. Fifth, the analytical sample is restricted to adults aged ≥ 65 years, such that these results cannot be extrapolated to younger age groups. Longitudinal cohorts covering all adult age ranges are needed to externally replicate these cross-sectional observations.

Key strengths of the present analysis cover a nationally representative sample of Chinese older adults, thorough adjustment for a broad set of confounders, and seven pre-specified sensitivity analyses that validated the stability of core associations. This work additionally yields preliminary evidence for effect modification across smoking subgroups among Chinese older adults, though this stratified observation awaits replication in larger independent cohorts.

## Conclusion

This cross-sectional analysis identified a statistically significant positive association between living within 300 m of major roadways and elevated diabetes prevalence among Chinese older adults. A statistically significant overall trend test was detected across ordered distance strata, yet the exposure–response curve followed a non-monotonic shape, with no steady stepwise elevation in diabetes prevalence as residential distance to roadways decreased. A borderline multiplicative interaction with smoking status was observed. Given the small sample volume within the smoking stratum and broad confidence intervals indicative of low estimation precision, this marginal subgroup signal merits cautious interpretation and replication in larger, statistically well-powered investigations. Crucially, roadway proximity functions as a composite contextual marker encapsulating traffic-derived hazards, neighborhood socioeconomic deprivation, and built environment attributes, instead of a standalone metric for one specific environmental exposure. Accordingly, the detected correlation cannot be solely ascribed to traffic-related air pollutants or road traffic noise.

These preliminary observational results prevent robust causal inferences and lack sufficient evidence to support urgent targeted policy interventions. Subsequent longitudinal studies integrating objective on-site environmental monitoring and granular neighborhood socioeconomic metrics are needed to replicate the current associations and inform evidence-based age-friendly urban design as well as community diabetes prevention programs tailored for older adults.

## Supplementary Information

Additional file 1: Full technical descriptions of the two FCS-MI approaches, model specifications, convergence evaluation metrics (FMI, RIV, RE), and comparative analyses against complete-case analysis.

Additional file 2: This table compares odds ratios for prevalent diabetes associated with residential proximity to major roadways across two regression models. Model 4 is fully adjusted for five health indicators (hypertension, anxiety symptoms, depression symptoms, activity limitation, self-reported quality of life), while Model 5 excludes these five health variables. Estimates including ORs, 95% CIs and *P*-values are provided for each roadway proximity category to illustrate how adjustment for physical and mental health indicators correlates with the observed association magnitudes.

Additional file 3: This table compares baseline demographic, behavioral, and metabolic characteristics between participants retained in the complete-case analysis (*n* = 4,702) and those excluded due to missing exposure or covariate data (*n* = 9,328), from the 14,030 participants with complete diabetes status. Means (SD) or percentages are presented for each variable, with P-values from t-tests or chi-square tests for group comparisons. This comparison evaluates potential selection bias related to missing data and helps interpret discrepancies between imputed and complete-case results.

Additional file 4: This table presents sensitivity regression results comparing associations between residential proximity to major roadways and prevalent diabetes under two distinct FCS-MI frameworks. Model 6: conventional FCS-MI (*n* = 15,874), with diabetes status, exposure and all covariates included in the imputation model. Model 7: outcome-fixed FCS-MI (*n* = 14,030), where only exposure and covariates with missing values are imputed among participants with complete diabetes outcome records. Estimates including ORs, 95% CIs and P-values are provided for each roadway proximity category. Imputation convergence metrics are also reported: Fraction of Missing Information (FMI), Relative Increase in Variance (RIV), and Relative Efficiency (RE). This table evaluates the stability of association estimates across alternative missing-data handling strategies.

Additional file 5: This table reports fully adjusted logistic regression estimates for the association between residential proximity to major roadways and prevalent diabetes across two sensitivity analyses. Panel A includes only urban and town residents (*n* = 2,871), with three roadway proximity groups (> 300 m as the reference: 100–300 m, < 100 m). Panel B uses five refined distance strata in the full complete-case sample (*n* = 4,702): ≤ 50 m, 50–100 m, 101–200 m, 201–300 m, and > 300 m as reference. ORs, 95% CIs and *P*-values are provided for every proximity subgroup.

## Data Availability

The data that support the findings of this study are available from the Chinese Longitudinal Healthy Longevity Survey (CLHLS). These data are not publicly available because access requires reasonable request and approval. The authors obtained approval to use the data for this study. Data may be available from the corresponding author upon reasonable request and with permission from the CLHLS.

## Abbreviations

HbA1c: Glycated Hemoglobin A1c
ADL: Activities of Daily Living
BMI: Body Mass Index
CES-D: Center for Epidemiologic Studies Depression
CI: Confidence interval
CLHLS: Chinese Longitudinal Healthy Longevity Survey
FCS-MI: Fully Conditional Specification Multiple Imputation
FMI: Fraction of Missing Information
GAD: Generalized Anxiety Disorder Scale
GLM: Generalized linear model
IDF: International Diabetes Federation
OR: Odds ratio
RE: Relative Efficiency
RIV: Relative Increase in Variance
SD: Standard deviation
