# Supplementary material for: Residential proximity to major roadways and prevalent diabetes in older adults: a cross-sectional study based on CLHLS
Source: BMC Geriatr. 2026 Jul 28;26:1325. doi: 10.1186/s12877-026-08060-y (PMC13685194; doi:10.1186/s12877-026-08060-y)
Supplement: Supplementary file 1 [file 12877_2026_8060_MOESM1_ESM.docx]

**Supplementary Methods: Multiple imputation details**

Two Fully Conditional Specification Multiple Imputation (FCS-MI) approaches with 20 iterations and 20 imputed datasets were implemented following the framework proposed by White et al. Convergence was verified using three imputation diagnostic metrics: Fraction of Missing Information (FMI), Relative Increase in Variance (RIV), and Relative Efficiency (RE). Consistent with standard missing-data guidelines, we predefined convergence thresholds of FMI < 0.3 and RIV < 1; we additionally applied a conservative cutoff of RE > 0.95 derived from Rubin’s rules, and no signs of non-convergence were detected across all imputed datasets.

Model 6 (conventional FCS-MI) incorporated the full initial cohort of 15,874 participants, jointly imputing missing values for exposure, covariates, and diabetes outcome. Model 7 (outcome-fixed FCS-MI) restricted the sample to 14,030 individuals with complete diabetes outcome records; only missing exposure and covariate values were imputed, and diabetes status was excluded from the imputation framework entirely.

Pooled logistic regression estimates generated from both FCS-MI models were compared against results from the primary complete-case analysis (n = 4,702) to assess bias attributable to missing data.
