# Supplementary material for: Residential proximity to major roadways and prevalent diabetes in older adults: a cross-sectional study based on CLHLS
Source: BMC Geriatr. 2026 Jul 28;26:1325. doi: 10.1186/s12877-026-08060-y (PMC13685194; doi:10.1186/s12877-026-08060-y)
Supplement: Supplementary file 2 [file 12877_2026_8060_MOESM2_ESM.docx]

Supplementary Table S1 Odds ratios for prevalent diabetes in relation to residential proximity to major roadways: comparison of Model 4 (primary fully adjusted model with five health indicators) versus Model 5 (model excluding the five health indicators)

| Model | Residential proximity to major roadways (m), OR (95% CI) | | | *P* for trend |
| --- | --- | --- | --- | --- |
|  | ＜ 100 | 100–300 | > 300 |  |
| Model 4 | 1.36 (1.11, 1.67) | 1.31 (1.03, 1.66) | Ref. | 0.004 |
| Model 5 | 1.32 (1.08, 1.61) | 1.33 (1.06, 1.68) | Ref. | 0.009 |

Note:

Model 4 represents the primary fully adjusted model incorporating five health-related intermediate indicators (hypertension, depressive symptoms, anxiety symptoms, activity limitation, self-reported quality of life).

Model 5 excludes the five above health indicators from the covariate set of Model 4 to preliminarily explore potential intermediate pathways between roadway proximity and prevalent diabetes.

All covariates other than the five health indicators remained consistent across Model 4 and Model 5.

Abbreviation: OR, odds ratio; CI, confidence interval; Ref., reference group.
