# Supplementary material for: Residential proximity to major roadways and prevalent diabetes in older adults: a cross-sectional study based on CLHLS
Source: BMC Geriatr. 2026 Jul 28;26:1325. doi: 10.1186/s12877-026-08060-y (PMC13685194; doi:10.1186/s12877-026-08060-y)
Supplement: Supplementary file 3 [file 12877_2026_8060_MOESM3_ESM.docx]

Supplementary Table S2 Baseline characteristics of participants retained for complete-case analysis versus those excluded owing to missing data

| Characteristics | Retained group (all variables complete) | Excluded group (intact diabetes outcome, missing covariates) | *P-*value |
| --- | --- | --- | --- |
| **N** | 4,702 | 9,328 |  |
| **Age, years, mean (SD)** | 78.7 ± 9.8 | 88.6 ±11.2 | < 0.001 |
| **BMI (kg/m^2^), mean (SD)** | 23.4 ± 6.6 | 22.1 ± 6.2^f^ | < 0.001 |
| **Residential proximity to major roadways, n (%)** |  |  | < 0.001 |
| < 100 m | 1,739 (37.0) | 2,587 (31.5) ^f^ |  |
| 100–300 m | 975 (20.7) | 1,543 (18.8) |  |
| > 300m | 1,988 (42.3) | 4,091 (49.8) |  |
| **Sex, *n* (%)** |  |  | < 0.001 |
| Male | 2,592 (55.1) | 3,601 (38.6) |  |
| Female | 2,110 (44.9) | 5,727 (61.4) |  |
| **Residential location, *n* (%)** |  |  | < 0.001 |
| City/Town | 2,871 (61.1) | 4,926 (52.8) |  |
| Rural | 1,831 (38.9) | 4,402 (47.2) |  |
| **Educational level, *n* (%)** |  |  | < 0.001 |
| No school | 1,158 (24.6) | 4,720 (65.3) ^f^ |  |
| ≥1 year | 3,544 (75.4) | 2,512 (34.7) |  |
| **Cohabitation status, *n* (%)** |  |  | < 0.001 |
| Yes | 3,930 (83.6) | 7,252 (79.3) ^f^ |  |
| No | 772 (16.4) | 1,897 (20.7) |  |
| **Self-reported quality of life, *n* (%)** |  |  | < 0.001 |
| Very good, good | 3,493 (74.3) | 5,540 (67.7) ^f^ |  |
| Average, bad, very bad | 1,209 (25.7) | 2,646 (32.3) |  |
| **Depressive** **symptoms ^a^, *n* (%)** |  |  | < 0.001 |
| Yes | 1,600 (34.0) | 3,075 (46.5) ^f^ |  |
| No | 3,102 (66.0) | 3,544 (53.5) |  |
| **Anxiety symptoms ^b^, *n* (%)** |  |  | < 0.001 |
| Yes | 450 (9.6) | 1,038 (12.7) ^f^ |  |
| No | 4,252 (90.4) | 7,131 (87.3) |  |
| **Cognitive impairment ^c^, *n* (%)** |  |  | < 0.001 |
| Yes | 300 (6.4) | 246 (10.5) ^f^ |  |
| No | 4,402 (93.6) | 2,107 (89.5) |  |
| **Smoking status, *n* (%)** |  |  | < 0.001 |
| Yes | 900 (19.1) | 1,177 (12.8) ^f^ |  |
| No | 3,802 (80.9) | 8,013 (87.2) |  |
| **Alcohol consumption, *n* (%)** |  |  | < 0.001 |
| Yes | 892 (19.0) | 1,115 (12.2) ^f^ |  |
| No | 3,810 (81.0) | 8,002 (87.8) |  |

**Supplementary Table S2 on next page**

**Supplementary Table S2 (continued)**

| Characteristics | Retained group (all variables complete) | Excluded group (intact diabetes outcome, missing covariates) | *P-*value |
| --- | --- | --- | --- |
| **Regular physical activity, *n* (%)** |  |  | < 0.001 |
| Yes | 2,598 (55.3) | 7,070 (77.4) ^f^ |  |
| No | 2,104 (44.7) | 2,066 (22.6) |  |
| **Activity limitation, *n* (%)** |  |  | < 0.001 |
| Yes | 994 (21.1) | 4,130 (44.5) ^f^ |  |
| No | 3,708 (78.9) | 5,148 (55.5) |  |
| **Annual household income (Yuan), *n* (%)** |  |  | < 0.001 |
| ≤ 10000 | 1,294 (27.5) | 2,970 (36.5) ^f^ |  |
| 10001–50000 | 1,519 (32.3) | 2,918 (35.9) |  |
| > 50000 | 1,889 (40.2) | 2,243 (27.6) |  |
| **Marital status, *n* (%)** |  |  | < 0.001 |
| Married | 2,867 (61.0) | 2,907 (31.6) ^f^ |  |
| Other categories ^d^ | 1,835 (39.0) | 6,284 (68.4) |  |
| **Hypertension, *n* (%)** |  |  | < 0.001 |
| Yes | 2,238 (47.6) | 3,387 (36.9) ^f^ |  |
| No | 2,464 (52.4) | 5,802 (63.1) |  |

Note:

Retained group, participants with complete data for diabetes outcome and all covariates (fixed n = 4,702).

Excluded group, participants with intact diabetes outcome but incomplete covariate data (total n = 9,328).

Parenthetical values within the excluded group column indicate valid non-missing participants for each characteristic.

All between-group comparisons yielded *P* < 0.001.

ᵃ Depressive symptoms were defined as a CES-D-10 total score of ≥ 10.

ᵇ Anxiety symptoms were defined as a GAD-7 total score of ≥ 5.

ᶜ Cognitive impairment was defined as a total cognitive function score of < 24.

ᵇ “Other categories” includes widowed, separated, divorced, and never-married participants.

ᵉ BMI, body mass index.

ᶠ Valid sample sizes differed across characteristics in the excluded group due to variable-specific missingness, all smaller than the total n = 9,328. Stratified valid sample counts are listed below:

BMI = 8,311; residential proximity to major roadways = 8,221; educational level = 7,232; cohabitation status = 9,149; self-reported quality of life = 8,186; depressive symptoms = 6,619; anxiety symptoms = 8,169; cognitive impairment = 2,353; smoking status = 9,190; alcohol consumption = 9,117; regular physical activity = 9,136; activity limitation = 9,278; annual household income = 8,131; marital status = 9,191; hypertension = 9,189.
