# Supplementary material for: Residential proximity to major roadways and prevalent diabetes in older adults: a cross-sectional study based on CLHLS
Source: BMC Geriatr. 2026 Jul 28;26:1325. doi: 10.1186/s12877-026-08060-y (PMC13685194; doi:10.1186/s12877-026-08060-y)
Supplement: Supplementary file 4 [file 12877_2026_8060_MOESM4_ESM.docx]

Supplementary Table S3 Sensitivity analyses of associations between residential proximity to major roadways and prevalent diabetes via two FCS-MI multiple imputation approaches

| Model | N | Residential proximity to major roadways (m) | OR (95% CI) | *P-*value | FMI | RIV | RE |
| --- | --- | --- | --- | --- | --- | --- | --- |
| Model 6 (conventional FCS-MI) | 15,874 | ＜ 100 | 1.31 (1.14, 1.50) | < 0.001 | 0.17 | 0.20 | 0.99 |
|  |  | 100–300 | 1.22 (1.04, 1.43) | 0.015 | 0.15 | 0.17 | 0.99 |
|  |  | > 300 (Ref.) | 1.00 | *—* | — | — | — |
|  | | | | | | | |
| Model 7  (outcome-fixed FCS-MI) | 14,030 | ＜ 100 | 1.28 (1.12, 1.47) | < 0.001 | 0.06 | 0.07 | 1.00 |
|  |  | 100–300 | 1.22 (1.04, 1.43) | 0.013 | 0.03 | 0.02 | 1.00 |
|  |  | > 300 (Ref.) | 1.00 | — | — | — | — |

Note:

Two FCS-MI multiple imputation sensitivity strategies were applied, corresponding to Model 6 and Model 7 respectively.

Model 6 (conventional FCS-MI): Conventional fully conditional specification imputation, with missing values for both covariates and residential proximity exposure imputed.

Model 7 (outcome-fixed FCS-MI): The binary prevalent diabetes outcome was retained as fixed intact data; only missing covariate values were imputed.

All imputed regression models adopted the covariate set of the primary fully adjusted Model 4.

Abbreviation: OR, odds ratio; CI, confidence interval; FMI, Fraction of Missing Information; RIV, Relative Increase in Variance; RE, Relative Efficiency; FCS-MI, Fully Conditional Specification Multiple Imputation.
