# Supplementary material for: Residential proximity to major roadways and prevalent diabetes in older adults: a cross-sectional study based on CLHLS
Source: BMC Geriatr. 2026 Jul 28;26:1325. doi: 10.1186/s12877-026-08060-y (PMC13685194; doi:10.1186/s12877-026-08060-y)
Supplement: Supplementary file 5 [file 12877_2026_8060_MOESM5_ESM.docx]

Supplementary Table S4 Fully adjusted logistic regression results for two sensitivity analyses linking residential proximity to major roadways and prevalent diabetes

| Residential proximity to major roadways (m) | N | OR (95% CI) | *P*-value |
| --- | --- | --- | --- |
| **Panel A. Urban and town residents only** |  |  |  |
| > 300 m | 983 | Ref. | — |
| 100–300 m | 703 | 1.40 (1.06, 1.85) | 0.017 |
| < 100 m | 1,185 | 1.39 (1.08, 1.78) | 0.010 |
| **Panel B. Five fine-grained distance subgroups** | | |  |
| > 300 m | 1,988 | Ref. | — |
| 201–300 m | 455 | 1.20 (0.88, 1.64) | 0.243 |
| 101–200 m | 520 | 1.40 (1.05, 1.86) | 0.022 |
| 50–100 m | 836 | 1.32 (1.03, 1.69) | 0.031 |
| < 50 m | 903 | 1.41 (1.10, 1.80) | 0.006 |

Notes:

All regression models adopted the covariate set of the primary fully adjusted Model 4.

Panel A restricted the analytical sample to participants residing in urban or town areas only, with > 300 m as the reference subgroup.

Panel B reclassified residential proximity into five fine-grained distance subgroups using the full analytical sample, with > 300 m as the reference subgroup.

Abbreviation: OR, odds ratio; CI, confidence interval; Ref., reference group.
